# Multi-Scale Characterization of Conventional and Immediate Dental Implant Systems

**DOI:** 10.3390/jfb15110317

**Published:** 2024-10-26

**Authors:** Seeun Mok, Mori E. Naftulin, Luiz Meirelles, Minji Kim, Jie Liu, Christine H. Lee, Hany A. Emam, Courtney A. Jatana, Hua-Hong Chien, Ching-Chang Ko, Do-Gyoon Kim

**Affiliations:** 1 Division of Orthodontics, College of Dentistry, The Ohio State University, Columbus, OH 43210, USA; mok.40@buckeyemail.osu.edu (S.M.); liu.7050@buckeyemail.osu.edu (J.L.); christinelee8711@gmail.com (C.H.L.); ko.367@osu.edu (C.-C.K.); 2 Division of Oral and Maxillofacial Surgery and Anesthesiology, College of Dentistry, The Ohio State University, Columbus, OH 43210, USA; naftulin.4@osu.edu (M.E.N.); emam.4@osu.edu (H.A.E.); jatana.5@osu.edu (C.A.J.); 3 Division of Restorative and Prosthetic Dentistry, College of Dentistry, The Ohio State University, Columbus, OH 43210, USA; meirelles.1@osu.edu; 4 Graduate School of Clinical Dentistry, Ewha Womans University, Seoul 07804, Republic of Korea; minjikim@ewha.ac.kr; 5 Division of Regenerative Sciences and Periodontology, College of Dental Medicine, Medical University of South Carolina, Charleston, SC 29425, USA; chienh@musc.edu

**Keywords:** dental implant, immediate implant, nanoindentation, micro-computed tomography, resonance frequency analysis

## Abstract

We hypothesized that the different post-implantation healing stages between the conventional and immediate implantations produce different amounts and tissue composition of the peri-implant bone. Thus, the objective of the current study was to examine whether the stability of dental implant systems is associated with characteristics of the interfacial bone area at different post-implanation healing periods. Mandibular molars were extracted from each beagle dog. After 10 weeks post-extraction, a screw-type titanium dental implant was placed in the molar location following a conventional dental implant (*Con*) procedure. Simultaneously, mandibular premolars were extracted and the same type of dental implant was placed in the distal site of the extracted premolar root following an immediate dental implant (*Imm*) procedure. The implant stability quotient (ISQ) values were not significantly different between *Con* and *Imm* groups at 0-, 3-, and 6-weeks post-implantation. However, 3D micro-computed tomography and 2D histological images confirmed that the *Imm* system had more gaps between the bone and implant than the *Con* system. On the other hand, the nanoindentation modulus value at the bone–implant interface was significantly higher for the *Imm* group than the *Con* group at both 3 weeks and 6 weeks post-implantation. The current results from multi-scale characterization suggest that the higher interfacial bone quality of the *Imm* system, despite its earlier post-implantation stage, plays a crucial role in maintaining stability comparable to that of the *Con* system.

## 1. Introduction

Dental implantation is one of the most successful treatment options to replace missing teeth [1,2]. It is estimated that more than 12 million dental implants are annually sold in the global market [3]. Conventional screw-type dental implantation consists of a two-stage surgical process [4,5,6]. The first stage involves extracting a tooth, followed by filling the extraction socket with new bone tissue. The new bone regeneration lasts longer than 2 months post-extraction in patients [7]. The second stage is to place the dental implant in the newly formed bone by drilling and tapping. Consequently, the dental implant is tailor-fit to the threaded bone, increasing bone–implant contact (BIC) [6,8]. However, the bone tissues adjacent to the dental implant are damaged during the vigorous surgical procedure of implantation, which triggers active bone remodeling and modeling at the bone–implant interface [9,10,11,12].

The damaged interfacial bone tissues are removed by bone resorption cells (osteoclasts). The bone-forming cells (osteoblasts) are coupled with the osteoclasts to regenerate new bone tissues at the resorbed interfacial sites [13,14]. Osteoblasts secrete collagen and minerals, which are the major components of bone tissue [15]. The mineralization rapidly increases during the short period of primary mineralization and then gradually progresses during the longer stage of secondary mineralization, occurring over approximately 7 and 73 days in human bone, respectively [16,17]. The bone turnover rate, which includes 30 days of bone resorption and 80 days of bone formation and mineralization in human bone, is estimated to be approximately 4.25 months. In comparison, this rate is about 3 months in canine models and 1.5 months in rabbit models [17,18,19]. The bone turnover due to active bone remodeling at the implant interface can continue up to 5 years, triggered by masticatory loading on the dental implant system in patients [20,21].

Primary stability of the dental implant system, which includes the implant and surrounding bone at the macro-level, is primarily determined by the amount of peri-implant bone and any interfacial damage immediately after implantation surgery [12,22]. At this initial stage, the material properties of the interfacial bone at the tissue level are maintained as the bone tissue composition has not yet been altered by bone remodeling. On the other hand, the secondary stability of the dental implant system is progressively achieved through the active bone turnover, which alters both quantity and quality (composition) of the peri-implant bone during the early post-implantation stage and through continuous daily masticatory loading [12,22]. The quantity of the interfacial bone has been assessed using the BIC that controls micro-motion between the bone and implant surface [10,18,23,24]. On the other hand, the amount of peri-implant bone is responsible for bearing loads at the macro-level. Histological analysis in animal models showed that the dimension of the bone–implant interface ranges from 300 μm up to 1 mm from the surface of the dental implant, where active bone remodeling following the initial damage was observed [18,24,25,26]. However, the characteristics of the interfacial bone within this range have not been fully assessed.

The dynamic bone turnover at the bone–implant interface produces heterogeneous bone characteristics at different time points. Different from the conventional two-stage dental implantation, immediate dental implantation has been suggested to place the implant immediately after tooth extraction [8,27,28]. As such, the treatment time and procedures of implantation are significantly reduced without the post-extraction healing periods [29]. We hypothesized that the different post-implantation healing stages between the conventional and immediate implant systems produce different amounts and tissue composition of the peri-implant bone. Thus, the objective of the current study was to examine whether the stability of dental implant systems is associated with characteristics of the interfacial bone area at different post-implantation healing periods. We assessed the stability of the dental implant system using multi-scale characterization with nanoindentation at the nano-level, micro-computed tomography (micro-CT) at the micro-level, and resonance frequency analysis (RFA) at the macro-level.

## 2. Materials and Methods

### 2.1. Animal Model

Four adult male beagles, weighing between 12 and 14 kg with fully erupted permanent dentition, were used in this study. The animal research protocol was approved by the Institutional Animal Care and Use Committee (IACUC) of the Ohio State University (2021A00000046).

### 2.2. Dental Implants

**Figure 1:**
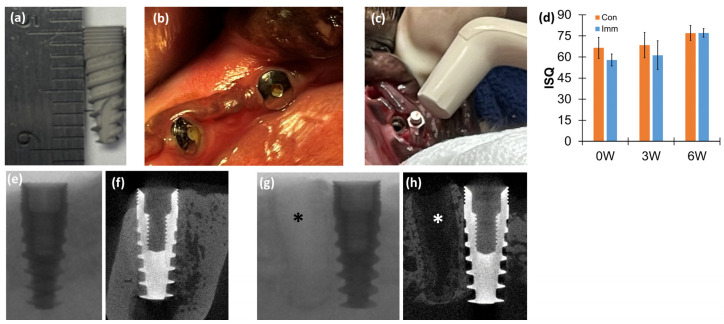
Implantation followed by resonance frequency analysis and computed tomography: (**a**) a titanium dental implant (ø3.75 × 10 mm) with sandblasted, large grit, acid-etched (SLA) surface treatment; (**b**) implantation sites after extraction of the second premolar and molar; (**c**) in vivo resonance frequency analysis (RFA); (**d**) not significantly different implant stability quotient (ISQ) between conventional dental implant (Con) and immediate dental implant (*Imm*) groups with 0-, 3-, and 6-weeks post-implantation periods (paired *t*-test, *p* > 0.078); (**e**) cone-beam tomography (CBCT) image scanned with 400-micron voxel size; (**f**) micro-tomography (micro-CT) image with 27-micron voxel size for Con; and (**g**) CBCT and (**h**) micro-CT images of *Imm* at 3 weeks post-implantation. * empty socket after tooth extraction. Each graph includes the average and standard deviation.

Conventional screw-type titanium dental implants (3.5 mm in diameter and 10 mm in length) with a large grit sandblast and acid-etched (SLA) surface (Dentin Implants Technologies, Ltd., Mizpe Aviv Industrial Park, Israel) were prepared (Figure 1a).

### 2.3. Surgical Procedure

All surgical procedures, anesthesia, and euthanasia adhered to methods approved by the American Veterinary Medical Association (AVMA) following the previous study [10,11]. Buprenorphine and meloxicam were administered at the time of surgery and again every 8 to 12 h for 3 days. Clindamycin (5 mg/kg, by mouth, every 12 h, for 5–8 days) was used for antibiotic treatment. After the surgeries, animals were fed a soft diet for 3 days followed by 1 to 2 days of weaning back to dry food and were monitored at least once every 24 h throughout the study. Sutures were removed 2 weeks after surgery. Animals were sedated with acepromazine and anesthetized with isoflurane and propofol. Pentobarbital overdose was used to euthanize the dogs.

All implantations followed the same protocol, without any chemical treatment except those required for the surgical process. Mandibular molars were extracted from each animal. Following positioning of jaw, muco-periosteal flaps were reflected on both sides to facilitate regular extraction of teeth in the mandible. The first molars and premolars were bilaterally extracted using a minimally traumatic technique, which involved sectioning the teeth along the furcation area with a bur to allow for individual root removal. After 10 weeks of post-extraction healing, dental implants were randomly placed in the molar position. Additionally, a second premolar was extracted to allow for the immediate placement of the same type of dental implant in the distal site of the extracted premolar root on the same day (Figure 1b,g,h). A 30-Newton-centimeter insertion torque was applied following the instructions for use provided by the manufacturer. A total of eight dental implants were placed bilaterally in the mandible of each animal. For this study, two implants were selected—one for immediate placement at the premolar position and the other for conventional placement at the molar position—both within the same hemi-mandible. The conventional implant was placed in the molar position because the molar, being the largest tooth, provides the most sufficient space for implantation once the bone has fully healed after extraction. In contrast, an immediate implant is placed right after tooth extraction, and the shape of the dental implant does not perfectly fit the irregular socket left after extraction. Therefore, a smaller extraction socket is preferred for immediate implantation. We chose the premolar position for the immediate implant because the premolar is close to the molar, offering similar bone properties, and its root is smaller than that of the molar.

### 2.4. Resonance Frequency Analysis (RFA)

Following implantation, resonance frequency analysis (RFA) was conducted for each dental implant (Figure 1c). Subsequently, a healing cap screw (3 mm cap height) was installed on each implant. For fluorescent bone labeling of newly formed bone, Alizarin red (30 mg/body weight (kg)) (Sigma, St. Louis, MO, USA) and Calcein green (10 mg/kg) (Sigma, St. Louis, MO, USA) were injected at 2- and 4-weeks post-implantation, respectively. The second RFA was performed for each dental implant system before 2 animals were sacrificed at both 3- and 6-weeks post-implantation, following the protocol established in the previous canine model for dental implantation [10].

The RFA probe (Ostell Mentor^®^, Osstell AB, Gothenburg, Sweden) was used to detect frequency signals from a resonance frequency sensor (SmartPeg^®^, Osstell AB, Gothenburg, Sweden) (Figure 1c). The proprietary implant stability quotient (ISQ) values provided by the RFA unit were utilized to quantify the stability of the dental implant system. An ISQ value for each dental implant system was obtained by averaging values measured in four directions (buccolingual and labiomesial and two reverse directions). Each individual dental implant system underwent RFA twice, immediately after implantation prior to installation of the healing screw and immediately after euthanasia.

### 2.5. Computed Tomography (CT)

**Figure 2:**
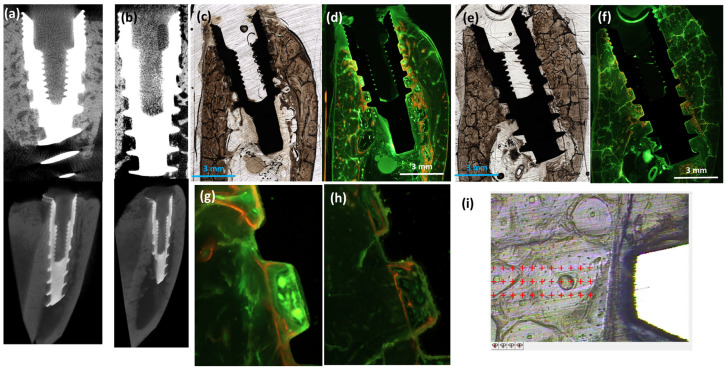
Three-dimensional micro-computed tomography and two-dimensional histology. Two-dimensional and three-dimensional high-resolution micro-CT images with 10-micron voxel size of (**a**) *Con* and (**b**) *Imm* as shown to expose the digitally dissected surface; (**c**,**e**) bright-field and (**d**,**f**) fluorescent images of *Con* and *Imm* at 6- and 3-weeks post-implantation, respectively; magnified fluorescent images of bone–implant interface for (**g**) *Con* and (**h**) *Imm*; and (**i**) 3 × 20 array with 30 μm between nanoindentations up to 600 μm from the implant surface. It was recommended to start the indentation at least 30 µm away from scratched areas next to the implant.

The bone–implant constructs were dissected in a hydrated state using a circulating diamond saw (EXAKT Technology, Oklahoma City, OK, USA). Subsequently, the specimen blocks were fixed in 10% formalin and dehydrated through a series of increasing concentrations of ethanol solutions. Finally, the specimen blocks were embedded in resin media and scanned by clinical cone-beam CT (CBCT) (Planmeca ProMax 3D, Saint Paul, MN, USA) with a voxel size of 400 μm (Figure 1e,f); micro-CT (Skyscan 1172D, Kontich, Belgium) with a voxel size of 27 μm (Figure 1g,h); and another micro-CT (μCT50, Scanco Medical AG, Wangen-Brüttisellen, Switzerland) with a voxel size of 10 μm (Figure 2a,b). The scanning conditions were CBCT (scanning energy (120 kV and 5 mA) and 8.9 s scanning time), Skyscan (70 kV, 141 μA, and 20 min scanning time), and μCT50 (70 kV, 114 μA, and more than 60 min scanning time).

### 2.6. Nanoindentation

The bone–implant constructs in the resin block were dissected in the buccal-lingual direction and their surfaces were polished for nanoindentation using a cutting saw and grinding system (EXAKT 400 CS Microgrinder, EXAKT Technology, Oklahoma City, OK, USA) under water irrigation. A total of 8 bone–implant constructs for 4 specimens of each conventional dental implant (Con) and immediate dental implant (*Imm*) groups at 3- and 6-weeks post-implantation were successfully prepared for nanoindentation (Figure 2i).

**Figure 3:**
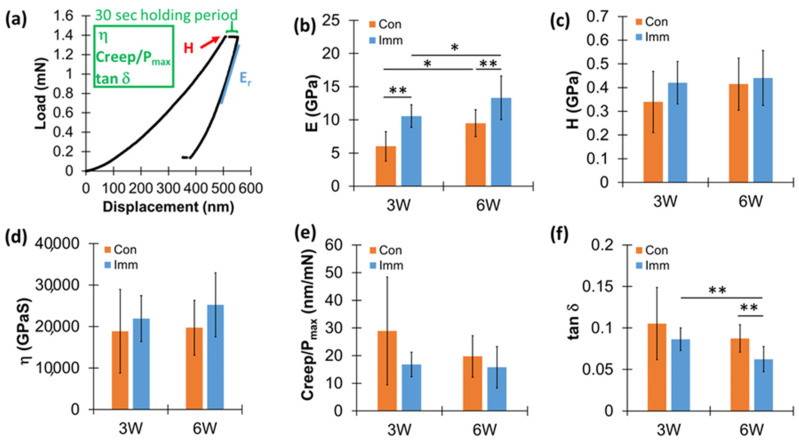
Comparison of nanoindentation parameters: (**a**) five nanoindentation parameters (reduced modulus (E_r_) that is converted to elastic modulus (E) [30], hardness (H), viscosity (η), Creep/P_max_, and tan δ) measured by a cycle of the indentation load–displacement curve at the same site. Comparisons of nanoindentation (**b**) modulus (E), (**c**) hardness (H), (**d**) viscosity (η), (**e**) Creep/Pmax, and (**f**) tan δ between *Con* and *Imm* implant groups at 3- and 6-weeks post-implantation. * *p* < 0.055, ** *p* < 0.03 (*t*-test). Each graph includes the average and standard deviation.

A detailed nanoindentation procedure was introduced in the previous study [30]. In brief, a pyramidal Berkovich tip was used to indent the interfacial bone next to the implant up to 500 nm deep with a displacement rate of 10 nm/sec (Figure 3a). The elastic modulus (E), plastic hardness (H), viscosity (η), normalized creep (Creep/P_max_), and tan δ were measured using a load–displacement curve at a peak indenting load (P_max_) during a 30-second hold period under peak load and unloading processes. The five parameters (E, H, η, Creep/P_max_, and tan δ) of elastic, plastic, and viscoelastic mechanical properties were obtained using a cycle of nanoindentation at the same location of the interfacial bone (Figure 3a). Indentations were performed in a 3 × 20 array of locations with 30 μm spacing between nanoindentations up to 600 μm from the implant surface in both buccal and lingual directions (Figure 2i). A total of 863 nanoindentations were successfully completed to provide valid values for analysis.

### 2.7. Histologic Examination

The specimen blocks were further ground less than 100 µm thickness using a cutting saw and grinding system (EXAKT 400 CS Microgrinder, EXAKT Technology, Oklahoma City, OK, USA) under water irrigation. Bright-field and fluorescent microscope images were taken using a BioTek Lionheart Lx Automated Microscope (Agilent, Santa Clara, CA, USA). Alizarin red and Calcein green fluorescent labels, used to identify newly formed bone tissues, were visualized through red and green filters of the fluorescent microscope at different time points (3 and 6 weeks) after implantation (Figure 2c–h).

### 2.8. Statistical Analysis

Early removal criteria were failure of healing of the muco-periosteal flap or infection of the bone that is not responsive to antibiotics/care as prescribed by the ULAR veterinary staff. No animals were removed. All data analyses were blinded, and JMP Pro 17 was used as the statistical software. A paired *t*-test was used to compare ISQ values of the *Con* and *Imm* groups between initial and post-implantation times. A *t*-test was performed to compare ISQ values and the nanoindentation modulus at each distance from the implant between the implant groups at each post-implantation time. Furthermore, a mixed model analysis of variance with individual implant as a random factor was performed to compare nanoindentation parameters between implant groups and post-implantation times. Significance was set at *p* < 0.05.

## 3. Results

The surgeries to extract teeth and place the dental implants were successfully conducted and no complications were observed during the post-implantation healing periods. The ISQ values were not significantly different between *Con* and *Imm* groups at 0-, 3-, and 6-weeks post-implantation (Figure 1d) (*p* > 0.078). The clinical CBCT image was unable to show the detailed structure of bone adjacent to the implant (Figure 1e) and provided a vague image of the extraction socket (Figure 1g), both of which were clearly distinguishable in the micro-CT images (Figure 1f,h).

The higher resolution (10-micron voxel size) micro-CT allowed 2D and 3D visualization of the bone–implant interface comparable to the 2D histology (Figure 2a–f). Both the 2D micro-CT and 3D histological images confirmed that the *Imm* system had more gaps between the bone and implant. The newly formed bone tissues adjacent to the implant at 6 weeks post-implantation were clearly labeled by the combination of Alizarin red and Calcein green labels injected at 2- and 4-weeks post-implantation, respectively (Figure 2d,g). The dynamic red and green color fluorescent microscopic images of the Con system indicate the active interfacial bone turnover. In contrast, the *Imm* system showed limited red-labeled interfacial bone tissues at 3 weeks post-implantation (Figure 2f,h).

The 3 × 20 array of nanoindentation sites was well-identified at the interfacial bone (Figure 2i). Nanoindentation modulus (E) values were significantly higher for the *Imm* group than the *Con* group at 3- and 6-weeks post-implantation (*p* < 0.023) and increased with post-implantation healing time (*p* < 0.055) (Figure 3b). Values of the hardness (H), viscosity (η), and Creep/P_max_ were not significantly different between implant groups and post-implantation periods (*p* > 0.078) (Figure 3c–e). The tan δ of the *Imm* group significantly decreased with post-implantation healing time (*p* < 0.001) and was lower for the *Imm* group than the *Con* group at 6 weeks post-implantation (*p* = 0.001) (Figure 3f).

**Figure 4:**
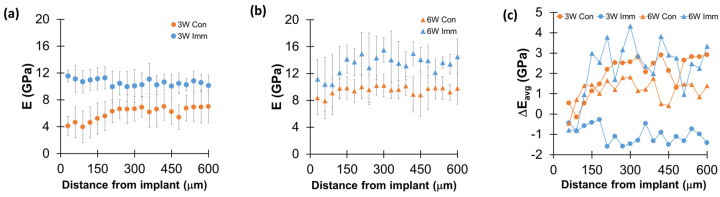
Immediate dental implant system had significantly higher nanoindentation modulus values than conventional dental implant system. Comparisons of the nanoindentation modulus (E) up to 600 μm distance from the surface of implant between *Con* and *Imm* groups at (**a**) 3 weeks and (**b**) 6 weeks post-implantation. Most E values were significantly higher for the *Imm* group than *Con* group at each distance (mixed-model ANOVA, *p* < 0.034). (**c**) The difference in average E (ΔE_avg_) between the first site (30 μm from the implant surface) and subsequent sites for all implant groups. (**a**,**b**) include the average and standard deviation.

All of the nanoindentation modulus (E) results with distance from the implant were significantly higher for the *Imm* group than the *Con* group at 3- and 6-weeks post-implantation (*p* < 0.034) (Figure 4a,b). The difference in average E (ΔE_avg_) between the first site (30 μm from the implant) and subsequent sites increased up to 300 μm for all implant groups, except the *Imm* group at 3 weeks post-implantation.

## 4. Discussion

The current findings indicate that the immediate dental implant (*Imm*) system had a comparable primary stability (ISQ value) to the conventional dental implant (Con) system, as assessed by resonance frequency analysis (RFA) at the macro-level. This result is consistent with observations from a previous clinical study [31]. However, 2D histology and 3D micro-CT images showed that the *Imm* system had more gaps between the implant and interfacial bone, which resulted in less peri-implant bone quantity than the *Con* system at the micro-level. Nevertheless, the *Imm* system maintained stronger bone quality adjacent to the implant than the *Con* system, as assessed by nanoindentation on the interfacial bone at the nano-level. This multi-scale characterization suggests that the higher interfacial bone quality of the *Imm* system, despite its earlier post-implantation stage, plays a crucial role in maintaining stability comparable to that of the *Con* system.

RFA has been used to assess the stability of dental implant systems with the bone–implant construct [11,32,33]. An implant stability quotient (ISQ) value of fifty or higher indicates that the dental implant system is mechanically stable enough for clinical use. RFA utilizes the resonance signal reflected from the smartpack connected to the abutment screw thread on top of the dental implant, allowing for the estimation of macro-level stability of the dental implant system. However, ISQ values can be influenced by various factors, including interfacial bone quantity (assessed by bone–implant contact area and interfacial dimensions) and quality (such as the degree of peri-implant bone mineralization and its material properties).

The immediate implantation after tooth extraction improves patient convenience due to fewer visits to the dental office; however, since the shape of the dental implant does not perfectly match the irregular socket after extraction, the *Imm* system often has more empty spaces compared to the *Con* system that is placed in a tailor-fit threaded site within fully healed bone. Consequently, the *Imm* system is expected to have a smaller bone–implant contact area than the *Con* system. A traditional methodology for measuring bone–implant contact is to use 2D histology of the dental implant system. However, the 2D image of the dissected surface may not completely illustrate the 3D architecture of the dental implant system, leading to bias in analysis depending on the location of dissection. On the other hand, CT provides a 3D volumetric image of the dental implant system. The current study showed various sizes of gaps between the implant and interfacial bone of the *Imm* system using both 2D histology and 3D high-resolution micro-CT imaging. However, the low resolution of the clinical CT scans was not sufficient to distinguish peri-implant bone from the metal implant. Increasing the resolution with micro-CT could provide detailed architecture of how the interfacial bone integrates with the dental implant and the shape of the socket next to the implantation site. Thus, the higher resolution scans improved the quality of the micro-CT images, similar to histological images. Despite its better quality, micro-CT-based 3D analysis of the bone–implant contact volume was not feasible due to the metal artifacts and coarse contrast between the metal implant and surrounding bone tissue. Further studies are needed using higher resolution or synchrotron micro-CT and advanced algorithms to better distinguish the metal implant from the surrounding bone [34].

The current study utilized nanoindentation to assess the elastic, viscoelastic, and plastic characteristics of bone tissues next to the implant. As the nanoindentation modulus (E) showed a significant and positive linear correlation with the degree of bone mineralization [35,36], a higher value indicates more mineralized bone tissue. Stiffer bone tissue has less ability to dissipate energy because the proportion of viscoelastic collagen is reduced between the dense minerals. We found that the E values were higher for the *Imm* group than the *Con* group and increased with post-implantation healing time. These findings indicate that the mineral contents in the interfacial bone tissue were higher for the *Imm* group than the *Con* group and progressive mineralization developed with increasing healing periods. These results are consistent with previous studies, which have shown that newly formed bone tissues adjacent to the implant have lower nanoindentation moduli than older, pre-existing bone tissues farther from the implant [37,38]. It was also found that newly formed bone within 150 μm of the implant had a significantly lower mineral-to-matrix ratio, crystallinity, and collagen cross-link ratio than mature bone located 300 μm from the implant after 10 weeks post-implantation [38]. These findings support our observation that the tan δ decreased with increasing mineralization over a longer post-implantation healing period, as tan δ decreases with a higher ratio of mineral-to-collagen matrix in bone.

However, fluorescent labels for newly forming bone tissues were not actively shown in the *Imm* system at 3 weeks post-implantation (Figure 2f). This result indicates that the interfacial bone of the *Imm* system was not dynamically remodeled yet, maintaining the pre-existing bone. These interfacial bone tissues of the *Imm* system may be damaged during rigorous implantation but sustain their mineral content higher than the newly formed interfacial bone tissues of the *Con* system, resulting in significantly higher E values. However, the mineralization process of the *Imm* system may accelerate with longer healing periods, resulting in significantly higher E values compared to the *Con* system and increase in E values with greater distances from the implant at 6 weeks post-implantation. These results provide insight that the interfacial gaps in the *Imm* system may facilitate more blood supply, which brings more bone cells, oxygen, and growth factors. This is consistent with findings from the previous canine study using a porous hybrid dental implant system [10].

We also found that the E values of the interfacial bone tissue increased up to 300 μm from the implant, indicating active turnover of the interfacial bone, except in the *Imm* system at 3 weeks post-implantation (Figure 4). This finding supports previous results, which estimated that active remodeling of the interfacial bone extends up to 300 μm from the implant [26,38]. This process involves the removal of damaged interfacial bone tissues and the addition of newly formed, less-mineralized bone tissues. Thus, this range of changes reflects the extent of bone damage caused by rigorous implantation surgery.

A limitation of the current study may be the small sample size. While the canine model is valuable due to its similarity to human bone, the number of dogs used in vivo experiments is limited by ethical concerns. Instead of increasing the number of dogs, we conducted additional investigations on the same specimens using multi-scale characterization techniques. Another limitation was that we did not use hydration conditions to conduct nanoindentation. Fixation and dehydration are inevitably required to proceed with a series of procedures for non-decalcified dissection of the bone–implant constructs. However, the dry condition of specimens may increase E values [39] and have a negative effect on the viscoelastic behavior of collagen components in the bone matrix. Therefore, caution should be exercised when comparing these measures with those from other studies.

## 5. Conclusions

The *Imm* system demonstrated comparable stability to the *Con* system. Results from multi-scale characterization indicate that, in the early stages post-implantation, the *Imm* system exhibited a smaller quantity of bone–implant interface compared to the *Con* system. However, the peri-implant bone quality in the *Imm* system was maintained as the material properties of the interfacial bone tissues were not altered prior to the onset of active bone remodeling triggered by surgical damage up to 300 µm from the implant surface. The rapid increase in peri-implant bone quality in the *Imm* system during the extended healing period may be attributed to sufficient blood supply to the empty gap between the implant and the interfacial bone. This study determined the interfacial bone dimensions and their associated multi-scale characteristics, which can provide baseline information for improving dental implant design, developing new implant materials, and enhancing finite element simulations to better assess the stability of implant systems, as suggested by previous studies [18,24,40,41,42].

## Data Availability

The original contributions presented in the study are included in the article, further inquiries can be directed to the corresponding author.
